# Daratumumab‐based induction and autologous transplantation in concomitant multiple myeloma and chronic myeloid leukemia

**DOI:** 10.1002/jha2.765

**Published:** 2023-09-11

**Authors:** Carmine Liberatore, Francesca Fioritoni, Annalisa Natale, Guido Montanaro, Gaetano La Barba, Cecilia Passeri, Ornella Iuliani, Bianca Fabi, Stefano Baldoni, Donatella Fantasia, Giuseppe Calabrese, Patrizia Accorsi, Stella Santarone, Stefano Pulini, Mauro Di Ianni

**Affiliations:** ^1^ Hematology Unit, Department of Oncology and Hematology Pescara Hospital Pescara Italy; ^2^ Bone Marrow Transplant Unit, Department of Oncology and Hematology Pescara Hospital Pescara Italy; ^3^ Blood Bank Unit, Department of Oncology and Hematology Pescara Hospital Pescara Italy; ^4^ Onco‐hematological Genetics, Department of Oncology and Hematology Pescara Hospital Pescara Italy; ^5^ Department of Medicine and Sciences of Aging University of Chieti‐Pescara Chieti Italy

**Keywords:** CML, multiple myeloma, myeloma, transplantation

## Abstract

The coexistence of chronic myeloid leukemia (CML) and multiple myeloma (MM) is a rare clinical condition. By means of FISH and molecular analysis on both sorted CD138 plasma cells and cryopreserved CD34 stem cells, a distinct clonal origin of the hematological malignancies was demonstrated in our case. We report on the first patient diagnosed with CML and MM treated with daratumumab, bortezomib, thalidomide, and dexamethasone (Dara‐VTd) induction, stem‐cell collection, and autologous stem cell transplantation (ASCT). The co‐administration of Dara‐VTd and imatinib proved feasible and highly effective in the management of both CML and MM. Despite concerns with stem cell mobilization and collection in patients exposed to daratumumab, in our experience the use of higher cyclophosphamide dose 4 g/m^2^ together with plerixafor granted optimal stem cell mobilization and collection, irrespective of daratumumab, concomitant myeloid neoplasm, and imatinib. Moreover, ASCT was easily performed with a rapid hematological reconstitution.

1

A 62‐year‐old woman presented with hypogammaglobulinemia, fatigue, and lower limb pain of recent onset. Twelve years before, she was diagnosed with chronic myeloid leukemia (CML) in chronic phase and treated with Imatinib. Major molecular response (MMR) was rapidly obtained and imatinib was prosecuted 200 mg once a day (QD).

At admission to our Institution, peripheral blood test showed anemia (hemoglobin 9.5 g/dL) and hypercalcemia (3.05 mmol/L). Serum creatinine was 76 mmol/L, lactate dehydrogenase (LDH) increased and beta2microglobuline 2.75 mg/L. Serum electrophoresis and immunofixation (IFX) found kappa light‐chain M‐protein (0.16 g/dL). Serum‐free light‐chains (FLC) kappa was 1630 mg/L and lambda 1.06 mg/L (FLC ratio 230.88). Urinary light‐chain kappa was 9730 mg/L and lambda less than 50 mg/L, whereas 24‐h urinary protein was 1618 mg. Bone marrow (BM) biopsy found atypical plasma cell (PCs) infiltration up to 50% of BM cellularity and complete remission (CR) of CML. Fluorescence in situ hybridization (FISH) on sorted CD138^+^ PCs found both gain 1q21 (three copies) and 14q32 rearrangement in 100% of analyzed cells, but no evidence of BCR::ABL1 fusion gene (1000 nuclei analyzed). BCR/ABL1 quantitative RT‐PCR (qRT‐PCR) on sorted PCs was also negative. Positron emission tomography‐computed tomography with ^18^FDG (PET TC) found multiple osteolytic lesions. Baseline BCR/ABL1 qRT‐PCR in peripheral blood (PB) was 0.389% on International Scale (IS) because of poor treatment compliance. Therefore, imatinib was increased up to 300 mg QD. The patient started induction therapy with daratumumab, bortezomib, thalidomide, and dexamethasone (Dara‐VTd). Treatment was well tolerated, without relevant toxicities. After first cycle, she obtained serum and urinary IFX negativity and FLC ratio normalization. After fourth cycle, stringent CR (sCR) was confirmed, with BM evaluation showing absence of PCs. BCR/ABL1 qRT‐PCR after fourth cycle were 0.0658% and 0.0629% in PB and BM, respectively. Thirty‐nine days after last daratumumab administration, the patient underwent stem‐cell mobilization with high‐dose cyclophosphamide (HD‐CTX; 4 g/m^2^), as per institutional practice [1]. Considering low collection amount (2.57 × 10^6^ CD34^+^ cells/kg) on first day of leukapheresis (Day +12), plerixafor 20 mg was prescribed. Thus, a total amount of 10.16 × 10^6^ CD34^+^ cells/kg was collected. The patient continued imatinib 300 mg QD during procedures. Cryopreserved CD34^+^ cells were examined: FISH analysis using BCR::ABL1, 1q21, and 14q32 rearrangement probes were negative (1000 nuclei analyzed), whereas BCR/ABL1 qRT‐PCR resulted positive (0.0640% IS). We tested 52 myeloid genes and 48 lymphoid genes by target sequences captured on custom NGS panels (Illumina), which found no mutations. After 6 months from diagnosis, she received conditioning with melphalan 200 mg/m^2^, followed by autologous stem cell transplantation (ASCT) with 4.93 × 10^6^ CD34^+^ cells/kg infusion. Stable neutrophils and platelets engraftments occurred after 10 and 19 days, respectively. No adverse events were reported. Imatinib was stopped at admission to the Transplantation Unit and resumed on Day +22. Two months later, the patient received two cycles of Dara‐VTd consolidation. Last disease assessment confirmed sCR of MM and MMR of CML (BCR/ABL1 qRT‐PCR 0.0059%). A PET TC showed complete metabolic response. Maintenance therapy with lenalidomide (10 mg QD) was started and imatinib (300 mg QD) prosecuted. The patient was treated according to current institutional programs upon written informed consent for transplantation procedures and use of medical records for research.

The coexistence of CML and MM is a rare clinical condition. To date, only 31 patients are reported: 12 had concurrent CML and MM, in 11 patients CML preceded MM, whereas in eight patients CML followed MM (Table 1). The interval between diagnosis is heterogeneous, spreading from months to years. Despite an origin of both myeloid and plasma cell malignancies from a common pluripotent hematopoietic progenitor is reasonable, the rarity and retrospective nature of reports did not allow to demonstrate a common origin. Schwarzmeier and colleagues clearly distinguished by means of FISH, the different natures of CML and MM [2]. The therapy‐related development of a second malignancy could also be a possibility, mainly in patients exposed to alkylating agents. Nonetheless, the incidental co‐occurrence of both malignancies remains the most reasonable explanation. In our case, the absence of BCR::ABL1 fusion gene on sorted PCs excluded a shared origin with CML. The presence of BCR/ABL1 fusion gene by qRT‐PCR on cryopreserved CD34^+^ cells suggested for the occurrence of CML funding lesion in a pluripotent hematopoietic progenitor. Conversely, the genesis of MM probably involved mature B cells, considering the negativity of 1q21 abnormalities and 14q32 rearrangement in cryopreserved CD34^+^ cells. Also, NGS proved a distinct clonal origin of MM and CML in our patient (Figure 1).

**TABLE 1:** Summary of main reports on patients with multiple myeloma and chronic myeloid leukemia.

Diagnosis	Reference	Year	Age at diagnosis	Sex	Interval between diagnosis	Treatment of CML	Treatment of MM
**Concomitant CML and MM**	Boots et al.; *J Clin Pathol*	1982	58	M		Busulfan, HU, thioguanine	RT + MP
	Tanaka et al.; *Acta Haematol*	1998	72	F		IFN alfa, vindesine, HU, steroids	–
	Alvarez‐Larrán et al.; *Haematologica*	2001	81	M		–	MP
	Schwarzmeier et al.; *Leukemia*	2003	66	M		HU, IFN alfa, busulfan	MP
	Wakayama et al.; *Med J Shimane Hosp*	2005	85	F		–	–
	Ide et al.; *Int J Hematol*	2010	72	F		Imatinib	–
	Offiah et al.; *Int J Hematol*	2012	71	F		Imatinib	MP, bortezomib, cyclophosphamide, Rd
	Ali et al.; *Hematol Rep*	2016	88	M		Imatinib	VRd
	Lee et al.; *Blood Res*	2017	64	M		–	TD, VAD, bortezomib
	Vishal et al.; *J R Coll Physicians Edinb*	2020	75	M		Imatinib	–
	Looi et al.; *Pathology*	2022	57	M		Imatinib	–
	Zhang et al.; *World J Clin Cases*	2022	48	M		Dasatinib	VRd
**CML preceding MM**	Derghazarian et al.; *CMAJ*	1974	65	F	9 years 5 months	Busulfan	RT + nitrogen mustards
	Yokota et al.; *Rinsho Ketsueki*	2005	71	M	3 years 2 months	Imatinib	–
	Garipidou et al.; *Oncologist*	2005	68	M	1 year 8 months	Imatinib	Melphalan, dexamethasone
	Ahn et al.; *Ann Lab Med*	2005	76	M	3 years	Imatinib	–
	Galanopoulos et al.; *Ann Hematol*	2009	76	M	1 year 2 months	Imatinib	TD
	Michalis et al.; *Oncologist*	2009	57	F	5 years 5 months	Imatinib	TD, VAD, bortezomib
	Pessach et al.; *Ann Hematol Oncol*	2015	63	F	6 years	Imatinib	Vd, Rd
	Katzel et al.; *Anti‐Cancer Drugs*	2015	63	M		Imatinib, nilotinib	Vd, Rd
	Swaminathan et al.; *F1000 Res*	2020	58	M	12 years	Imatinib	VTD
	McCaughan et al.; *Bone Marrow Transplant*	2021	65	M	4 years	Nilotinib	VCD + ASCT melphalan 200
	McCaughan et al.; *Bone Marrow Transplant*	2021	52	M	15 years	Imatinib	VCD + ASCT melphalan 200
	Liberatore et al.; *present case*	2023	62	F	12 years	Imatinib	DaraVTd + ASCT melphalan 200
**MM preceding CML**	MacSween et al.; *CMAJ*	1972	77	M	2 years 9 months	6‐MP	–
	Klenn et al.; *Yonsei Med J*	1993	71	M	2 years	HU	MP + RT
	Nitta et al.; *Int J Haematol*	1999	70	M	2 years 9 months	–	–
	Nakagawa et al.; *J Obihiro Kosei Gen Hosp*	2003	47	M	2 years 9 months	–	–
	Loheetha et al.; *Clin Lymph Myeloma Leuk*	2013	62	F	1 year 5 months	Dasatinib	RT, VCD, VCD + doxorubicin, VRD
	Alsidawi et al.; *Case Rep Oncologic Med*	2014	60	M	4 years	Dasatinib	Rd, Vd
	Pessach et al.; *Ann Hematol Oncol*	2015		M	4 years 7 months	Imatinib	VAD
	Miki et al.; *Case Rep Hematol*	2018	76	M	2 years	Dasatinib, bosutinib	Vd, VCD, Rd
	Yeung et al.; *Pathology*	2020	68	M	2 years	Imatinib	CTD

Abbreviations: 6‐MP, 6‐mercaptopurine; ASCT, autologous stem‐cell transplantation; DaraVTd, daratumumab bortezomib thalidomide dexamethasone; HU, hydroxyurea; IFN, interferon; MP, melphalan prednisone; Rd, lenalidomide dexamethasone; RT, radiotherapy; TD, thalidomide dexamethasone; VAD, vincristine doxorubicin dexamethasone; VCD, bortezomib, cyclophosphamide, dexamethasone; cyclophosphamide thalidomide dexamethasone; Vd, bortezomib dexamethasone; VRD, bortezomib lenalidomide dexamethasone.

**FIGURE 1:**
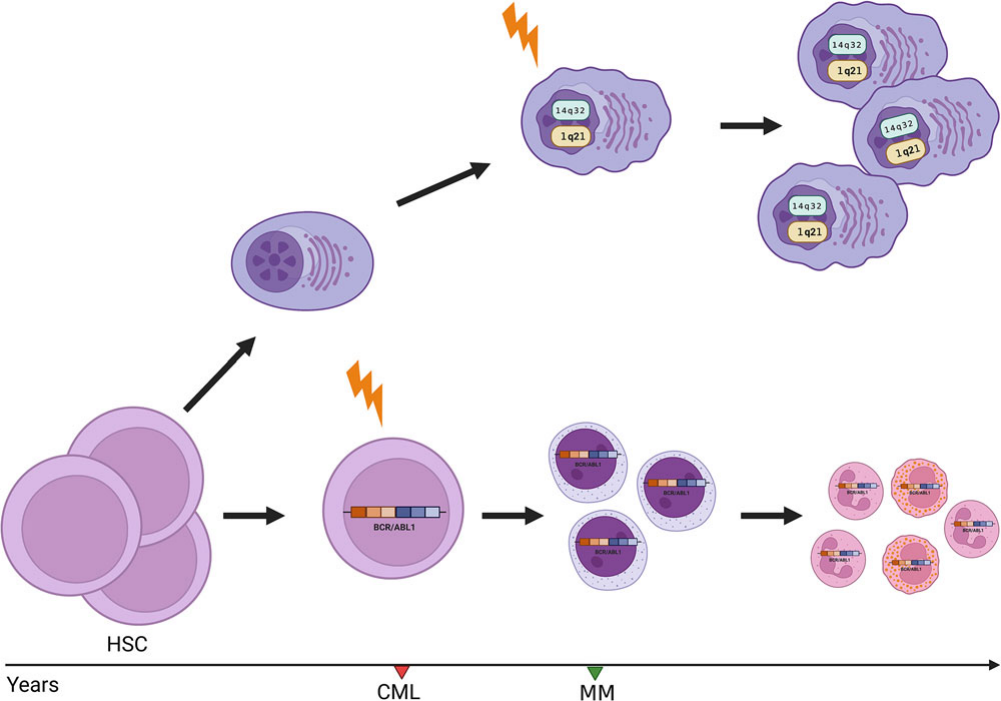
The proposed clonal evolution of multiple myeloma and chronic myeloid leukemia in our patient. In the lower side of the figure, the occurrence of BCR::ABL1 in a pluripotent hematopoietic progenitor originated the chronic myeloid leukemia. In the upper side of the figure, the occurrence of 1q21 abnormalities and 14q32 rearrangement in a mature plasma cell originated multiple myeloma. HSC: hematopoietic stem cell. Created with biorender.com.

Imatinib is the most widely reported treatment among patients with CML and MM, although second‐generation tyrosine kinase inhibitors have been used more recently. Addressing myeloma, the majority of patients were not eligible for intensive treatment due to advanced age and comorbidities (Table 1). Recently, McCaughan and colleagues reported on two patients with CML on imatinib and MM successfully treated with cyclophosphamide, bortezomib, and dexamethasone induction and ASCT [3].

To the best of our knowledge, we report on the first patient diagnosed with CML and MM treated with daratumumab‐based induction, stem‐cell collection, and ASCT. Co‐administration of imatinib and Dara‐VTd proved feasible, with limited hematological and nonhematological toxicities. Since Dara‐VTd has become the standard treatment for transplant‐eligible newly diagnosed multiple myeloma (NDMM) in Europe, concerns with stem‐cell mobilization emerged in patients exposed to daratumumab [4]. Following cyclophosphamide 2–3 g/m^2^ and G‐CSF in CASSIOPEIA trial, patients experienced greater use of plerixafor, lower collection of CD34^+^ cells/kg, and longer intervals to hematologic engraftment after ASCT [4]. Similar results are reported in real‐life reports with cyclophosphamide and with chemo‐free mobilization approach [5, 6, 7]. In our experience, higher cyclophosphamide dose 4 g/m^2^ together with plerixafor granted optimal stem‐cell collection [1]. Neither concomitant myeloid neoplasm nor the administration of daratumumab and imatinib impaired leukapheresis and ASCT.

In conclusion, CML and MM appeared as distinct entities in our case. The co‐administration of both Dara‐VTd and imatinib was well tolerated and highly effective in the management of both hematological malignancies. HD‐CTX 4 g/m^2^ and plerixafor granted optimal stem‐cell collection irrespective of daratumumab exposure and concomitant myeloid neoplasm, whereas ASCT was easily performed with a rapid hematological reconstitution.

## AUTHOR CONTRIBUTIONS

All co‐authors (Carmine Liberatore, Francesca Fioritoni, Annalisa Natale, Guido Montanaro, Gaetano La Barba, Cecilia Passeri, Ornella Iuliani, Bianca Fabi, Stefano Baldoni, Donatella Fantasia, Giuseppe Calabrese, Patrizia Accorsi, Stella Santarone, Stefano Pulini, Mauro Di Ianni) contributed to patients’ clinical care. Carmine Liberatore wrote the manuscript. Mauro Di Ianni revised the manuscript. All authors have read and agreed to the published version of the manuscript.

## CONFLICT OF INTEREST STATEMENT

The authors declare they have no conflicts of interest.

## CLINICAL TRIAL REGISTRATION

The authors have confirmed clinical trial registration is not needed for this submission.

## ETHICS STATEMENT

The authors have confirmed ethical approval statement is not needed for this submission.

## PATIENT CONSENT STATEMENT

The authors have confirmed patient consent statement is not needed for this submission.

## FUNDING INFORMATION

No financial support was received for this article.

## Data Availability

The data that support the findings of this study are available from the corresponding author upon reasonable request.
